# Ciprofloxacin-Induced Anaphylactic Reaction Followed by Negative Provocation Test in Response to Levofloxacin: A Case Report

**DOI:** 10.3390/medicina59101784

**Published:** 2023-10-07

**Authors:** Marija Kurtov, Paula Kilić, Lucija Ikić, Karlo Kurtov, Gordan Dorčić, Marko Vodanović, Marinko Artuković, Marina Ikić Matijašević

**Affiliations:** 1 Department of Clinical Pharmacology and Toxicology, University Hospital Sveti Duh, 10000 Zagreb, Croatia; 2 Department of Clinical Immunology, Rheumatology, and Pulmonology, University Hospital Sveti Duh, 10000 Zagreb, Croatia; paulakilic@gmail.com (P.K.); marinko.artukovic@gmail.com (M.A.); 3 Department of Anatomy and Physiology, University of Applied Health Sciences, 10000 Zagreb, Croatia; lucijaikic1995@gmail.com; 4 Department of Nephrology and Dialysis, University Hospital Merkur, 10000 Zagreb, Croatia; karlo.kurtov@gmail.com; 5 Department of Nephrology and Dialysis, University Hospital Sveti Duh, 10000 Zagreb, Croatia; g.dorcic@gmail.com; 6 Division of Gastroenterology and Hepatology, University Hospital Sveti Duh, 10000 Zagreb, Croatia; markovod@gmail.com; 7 Department of Internal Medicine, School of Medicine, University of Zagreb, 10000 Zagreb, Croatia

**Keywords:** fluoroquinolones, cross-reactivity, allergy, ciprofloxacin, levofloxacin, drug provocation test, skin prick test

## Abstract

Fluoroquinolones are a commonly prescribed class of antibiotics due to their broad spectrum of antimicrobial activity, favorable pharmacokinetic properties, ability to switch from parenteral to oral administration, and global availability. After beta-lactams, they are the second most common antibiotic class associated with drug allergies. The mechanism of fluoroquinolone-induced hypersensitivity reactions has not yet been fully understood, so the true incidence of hypersensitivity reactions remains unknown. Cross-reactivity between fluoroquinolones has been the subject of conflicting and limited clinical research. Due to their similar chemical structure, some argue for close cross-reactivity within the group. However, recent studies have produced contradictory results. We present the case of a young patient who had an anaphylactic reaction to ciprofloxacin but was tolerant to levofloxacin, as determined via a skin prick test followed by a drug provocation test. Our findings support the notion that there is little cross-reactivity between fluoroquinolones. Consequently, exposure to another fluoroquinolone in a hospital setting may be beneficial, particularly for patients who lack adequate antibiotic alternatives. However, additional research on this subject is required.

## 1. Introduction

Due to their broad spectrum of antimicrobial activity, favorable pharmacokinetic properties, ability to switch from parenteral to oral administration, and global accessibility, fluoroquinolones are a frequently prescribed class of antibiotics [1]. Fluoroquinolones exert their antimicrobial activity by interfering with DNA replication in Gram-positive and Gram-negative bacteria [2]. Therefore, they are used to treat a wide variety of infections, including urinary, respiratory, gastrointestinal, and intraabdominal infections, as well as sexually transmitted diseases [3,4]. Currently, the most commonly prescribed fluoroquinolones in our country are ciprofloxacin, levofloxacin, and moxifloxacin. As with other medications, adverse reactions, such as hypersensitivity reactions (HRs), have been reported with fluoroquinolone use. Specifically, they are the second most common class of antibiotics associated with drug allergies, after beta-lactams [1]. Since the mechanism of fluoroquinolone-induced hypersensitivity reactions has not yet been fully understood, the true incidence of HRs remains unknown. HRs to fluoroquinolones are classified as either immediate or delayed based on the time between exposure and the onset of the reaction. Immediate reactions occur more frequently than delayed ones [5,6]. Fortunately, anaphylactic reactions are uncommon, with an estimated incidence of 0.5–1.2 cases per 100,000 patients, and they are primarily reported during the post-marketing phase [7,8]. We present the case of a young patient who developed an anaphylactic reaction to ciprofloxacin, and nine months later, demonstrated tolerance to levofloxacin via a drug provocation test (DPT).

## 2. Detailed Case Description

A 33-year-old male patient was prescribed peroral ciprofloxacin after being in contact with a patient who had a confirmed *Neisseria meningitidis* infection. Three years prior to this event, our patient was treated for an intra-abdominal infection with ciprofloxacin without developing HR. With re-exposure to ciprofloxacin, the patient experienced abdominal pain, nausea, and the urge to vomit immediately after taking ciprofloxacin. Ten minutes later, he developed urticaria of the face, extremities, and trunk; diffuse erythema; itching; angioedema of the lips; and weakness without respiratory symptoms or hypotension. After observing an urticarial rash, treatment for an anaphylactic reaction was initiated in our patient, a hospital worker who was working at the time of drug administration. Infusions of crystalloid solutions, an H2-receptor blocker, chloropyramine, and intravenous methylprednisolone were administered to treat the anaphylactic reaction. Within 90 min, all his symptoms resolved, and he was admitted to the hospital and observed for 24 h without reoccurrence of the symptoms. Unfortunately, a lack of reagents prevented the testing of serum tryptase, and the serum sample itself was not stored. Prior to that, the patient had not had any drug-related allergic reactions. The patient’s medical history included multiple hospital admissions for severe necrotizing pancreatitis, which required four surgical interventions. In addition, he had allergic asthma, Gilbert syndrome, and type 2 insulin-dependent diabetes, and was taking empagliflozin, metformin, esomeprazole, escitalopram, cholecalciferol, and insulin aspart and degludec, and was undergoing pancreatic enzyme replacement therapy. Considering his medical history, it was highly probable that he would require fluoroquinolone treatment in the future. After discussion and in agreement with the patient, we decided nine months after ciprofloxacin anaphylaxis to perform a DPT for another quinolone, levofloxacin. The provocation test was performed in controlled hospital conditions under the supervision of trained staff. In vitro basophil activation tests (BAT) and immunoassays for detecting quinolone-specific IgE are not available in our country, so they were not performed before the in vivo tests. After obtaining the patient’s informed consent, a skin prick test (SPT) for levofloxacin with positive and negative controls (Table 1) was performed. An intradermal test (IDT) for levofloxacin was not performed due to our previous experience with irritative reactions with fluoroquinolones. We decided against conducting skin tests for ciprofloxacin since our patient presented with symptoms that were highly indicative of an immediate hypersensitivity reaction. After a negative SPT, we performed an oral provocation test for levofloxacin: a gradual dose increase (125–125–250 mg) in 60 min intervals until the full therapeutic dose was reached (Table 1). The patient was observed for an additional 24 h and, subsequently, after discharge, did not report any drug-related reactions. Therefore, the DPT for levofloxacin was considered negative, with a conclusion that it is safe for the patient to take levofloxacin in the future.

## 3. Discussion

The complete mechanism of HRs to fluoroquinolones is unknown, as there is evidence of both IgE-mediated and non-IgE-mediated immediate reactions [1,6,9]. In a type I immediate HR, antigen exposure initiates an immune response that results in the production of IgE antibodies, and re-exposure to the same antigen induces the degranulation of IgE-bound mast cells and basophils as well as the release of inflammatory mediators [10]. However, some low-molecular-weight drugs can induce an immune response through the haptenization of larger proteins [10]. It is believed that intermediates of partially metabolized fluoroquinolones may produce this type of immune response [1]. The direct activation of the Mas-related G-protein-coupled receptor member X2 (MPGPRX2) receptor of mast cells, completely independent of the activation of IgE antibodies, is an additional mechanism underlying the immediate reaction. In contrast to IgE-mediated reactions, these reactions are dose-dependent and can occur even upon first exposure to the drug [11]. Symptoms of immediate hypersensitivity reactions typically manifest in less than one hour but may occur six hours after exposure to fluoroquinolones. Symptoms include itching; dizziness or lightheadedness; nausea; chest pain; generalized erythema; urticaria or angioedema; or respiratory (dyspnea, wheezing, stridor, hypoxemia), cardiovascular (hypotension, collapse, altered conscious state, incontinence), or gastrointestinal (cramping, abdominal pain, vomiting) symptoms. In most cases, immediate-type hypersensitivity can be diagnosed clinically; however, if typical symptoms are absent, serial measurements of mast cell tryptase can help confirm the diagnosis [5]. Although serum tryptase, in vitro radioimmunoassay, and a BAT for ciprofloxacin were not performed in our patient, our patient had an immediate HR, most likely an IgE-mediated type I allergic reaction. Note that our patient has been exposed to ciprofloxacin once and has a medical history of allergic diseases, but no known drug allergies. Currently, validated commercial in vitro tests (radioimmunoassays and the BAT) for the evaluation of quinolone allergy for routine clinical use are neither widely available nor available in our country. Skin prick tests and IDTs are frequently used in the diagnostic workup of IgE-mediated immediate HRs to antibiotics, but their value in evaluating fluoroquinolone allergy is questionable since some fluoroquinolones can induce false-positive results due to their skin-irritant effect. Standardized allergens for SPT, such as those for beta-lactams, are unavailable. For SPT and IDT, only commercially available drugs in various non-irritating concentrations are used. In general, skin tests and in vitro tests have demonstrated low sensitivity and specificity; therefore, the DPT is considered the test of choice to confirm or exclude a quinolone allergy. However, it carries the risk of an allergic reaction, particularly anaphylaxis, in patients with a history of anaphylaxis to a drug from the same group [1,12,13,14]. Due to a history of anaphylaxis to one quinolone, ciprofloxacin, we performed an SPT with another quinolone, levofloxacin, to at least partially rule out cross-reactivity. Cross-reactivity between fluoroquinolones has been the subject of conflicting and limited clinical research. Due to their similar chemical structures, most studies assert that there is close cross-reactivity within the group. All fluoroquinolones have a bicyclic core structure with a fluorine atom at the C-6 position, but the structure of groups bound in the C1, C5, C7, and C8 positions may also be responsible for similar immune responses. Therefore, some authors strongly recommend avoiding all other fluoroquinolones in patients with an allergy to one fluoroquinolone, especially those with a history of severe HRs [15,16,17]. However, the following studies have contradictory findings. In a 2010 article by Chang B. et al., patients with a history of immediate HRs to moxifloxacin were re-exposed to the drug and exposed to ciprofloxacin. All three patients developed similar symptoms to those experienced upon previous exposure to moxifloxacin, confirming their allergy to moxifloxacin, but they all tolerated ciprofloxacin [18]. Another study examined 12 patients who had experienced an immediate reaction (four anaphylaxis and eight urticaria/angioedema) to oral quinolone administration. Most ciprofloxacin-reactive patients tolerated levofloxacin, while the majority of levofloxacin-reactive patients also tolerated ciprofloxacin. In addition, moxifloxacin-sensitive patients tolerated ciprofloxacin and levofloxacin. This study highlighted a lack of cross-reactivity among quinolones and suggested that levofloxacin may be a safer alternative to first-, second-, and fourth-generation quinolones [13]. In addition, Azimi et al. published a retrospective study on fluoroquinolone cross-reactivity. This study included 321 cases, 310 of which had a history of an immediate hypersensitivity reaction to ciprofloxacin, levofloxacin, and/or moxifloxacin. All participants were exposed to a second fluoroquinolone, but only eight participants experienced an immediate hypersensitivity reaction, yielding a cross-reactivity frequency of 2.5%. Only four out of 157 participants with a history of immediate HRs to ciprofloxacin developed an HR with levofloxacin exposure [19]. In addition, according to a case report from Japan, a 26-year-old man developed anaphylactic shock after receiving levofloxacin treatment. After he had fully recovered, a skin scratch test revealed a positive result for levofloxacin but a negative result for garenoxacin. The patient then tolerated the full therapeutic dose of garenoxacin [20]. Shah et al.’s findings from a recent large, multicenter, retrospective study suggest that patients with confirmed ciprofloxacin, moxifloxacin, or levofloxacin HRs may tolerate other fluoroquinolones. Seventy percent of patients in the study were challenged with a different fluoroquinolones, but only nine (5.6%) patients experienced hypersensitivity [21]. Similarly to our patient, a case report published in Elsevier Espana in 2012 presents a patient who developed an anaphylactic reaction to levofloxacin, but tolerated ciprofloxacin four weeks afterwards [22]. Furthermore, the cross-sectional study by Demir et al. included 54 patients with a history of 57 hypersensitivity reactions. Cross-reactivity was primarily observed between levofloxacin and ofloxacin (50.0%), whereas it occurred least between moxifloxacin and the other fluoroquinolones [23]. We chose DPT with levofloxacin for our patient based on previously mentioned studies demonstrating a lack of cross-reactivity between different quinolones and keeping in mind the patient’s possible future need for a quinolone-based medication. Because our patient had a severe HR to ciprofloxacin, we performed an SPT for levofloxacin rather than a direct oral test, thus excluding cross-reactivity between the two quinolones to a certain extent, as also suggested by some other authors; however, keep in mind that the results of some studies do not support the usefulness of SPTs in the diagnosis of a hypersensitivity reaction to quinolones, as well as in the prediction of cross-reactivity [13,22,23]. In the future, this could be the standard of care for patients with an immediate, especially severe, HR to a quinolone, with the goal of allowing for the safe use of another quinolone, but additional research is required.

## 4. Conclusions

The drug challenge test is still the gold standard for determining immediate HRs to fluoroquinolones. Further research is required on the mechanism of immediate HRs to fluoroquinolones. A better understanding of the underlying cascade of HRs could lead to the discovery of an appropriate test for fluoroquinolone allergy, which could explain the contradictory clinical experience regarding fluoroquinolone cross-reactivity. Until then, it is important to keep in mind that in vivo exposure to another fluoroquinolone in a hospital setting for patients with a history of allergy to one fluoroquinolone may be useful, particularly for patients without an adequate antibiotic alternative.

## Figures and Tables

**Table 1:** Protocol used for levofloxacin allergy testing in a patient with a ciprofloxacin-anaphylactic reaction. Skin prick test followed by an oral provocation test with levofloxacin (60 min interval between each step in the drug provocation test).

Step	SPT	DPT
	Drug concentration	Full dose: 500 mg
1.	1:10 (0.5 mg/mL)	¼ of full dose (125 mg)
2.	1:1 (5 mg/mL)	¼ of full dose (125 mg)
3.	-	½ of full dose (250 mg)

SPT—skin prick test; DPT—drug provocation test.

## Data Availability

The original data generated and analyzed for this study are included in the published article.

## References

[1] McGee E.U., Samuel E., Boronea B., Dillard N., Milby M.N., Lewis S.J. (2019). Quinolone Allergy. Pharmacy.

[2] Blondeau J.M. (2004). Fluoroquinolones: Mechanism of Action, Classification, and Development of Resistance. Surv. Ophthalmol..

[3] Wolfson J.S., Hooper D.C. (1991). Pharmacokinetics of Quinolones: Newer Aspects. Eur. J. Clin. Microbiol. Infect. Dis..

[4] Andriole V.T. (1999). The Future of the Quinolones. Drugs.

[5] Doña I., Blanca-López N., Boteanu C., Cueva-Oliver B., Fernández-Sánchez F., Gajate P., García-Avilés M., García-Núñez I., Lobera T., Moreno E. (2021). Clinical Practice Guidelines for Diagnosis and Management of Hypersensitivity Reactions to Quinolones. J. Investig. Allergol. Clin. Immunol..

[6] Blanca-López N., Andreu I., Torres Jaén M.J. (2011). Hypersensitivity Reactions to Quinolones. Curr. Opin. Allergy Clin. Immunol..

[7] Johannes C.B., Ziyadeh N., Seeger J.D., Tucker E., Reiter C., Faich G. (2007). Incidence of Allergic Reactions Associated with Antibacterial Use in a Large, Managed Care Organisation. Drug Saf..

[8] Portilho N.C., Aun M.V., Kalil J., Giavina-Bianchi P. (2020). Quinolone-Induced Anaphylaxis. Curr. Treat. Options Allergy.

[9] Doña I., Moreno E., Pérez-Sánchez N., Andreu I., Hernández Fernandez De Rojas D., Torres M.J. (2017). Update on Quinolone Allergy. Curr. Allergy Asthma Rep..

[10] Stone S.F., Phillips E.J., Wiese M.D., Heddle R.J., Brown S.G.A. (2014). Immediate-Type Hypersensitivity Drug Reactions: Immediate-Type Hypersensitivity Drug Reactions. Br. J. Clin. Pharmacol..

[11] Giavina-Bianchi P., Gonçalves D.G., Zanandréa A., Borges De Castro R., Garro L.S., Kalil J., Castells M. (2019). Anaphylaxis to Quinolones in Mastocytosis: Hypothesis on the Mechanism. J. Allergy Clin. Immunol. Pract..

[12] Perez E., Callero A., Martinez-Tadeo J.A., Hernandez G., Rodríguez-Plata E., Almeida Z., Garcia-Robaina J.C. (2013). Are Skin Tests Useful in Fluoroquinolone Hypersensitivity Diagnosis?. Ann. Allergy Asthma Immunol..

[13] Lobera T., Audícana M., Alarcón E., Longo N., Navarro B., Muñoz D. (2010). Allergy to Quinolones: Low Cross-Reactivity to Levofloxacin. J. Investig. Allergol. Clin. Immunol..

[14] Seitz C.S., Bröcker E.B., Trautmann A. (2009). Diagnostic Testing in Suspected Fluoroquinolone Hypersensitivity. Clin. Exp. Allergy.

[15] Dávila I., Diez M.L., Quirce S., Fraj J., Hoz B., Lazaro M. (1993). Cross-Reactivity between Quinolones: Report of Three Cases. Allergy.

[16] González I., Lobera T., Blasco A. (2005). Immediate Hypersensitivity to Quinolones: Moxifloxacin Cross-Reactivity. J. Investig. Allergol. Clin. Immunol..

[17] Anovadiya A.P., Barvaliya M.J., Patel T.K., Tripathi C.B. (2011). Cross Sensitivity between Ciprofloxacin and Levofloxacin for an Immediate Hypersensitivity Reaction. J. Pharmacol. Pharmacother..

[18] Chang B., Knowles S.R., Weber E. (2010). Immediate Hypersensitivity to Moxifloxacin with Tolerance to Ciprofloxacin: Report of Three Cases and Review of the Literature. Ann. Pharmacother..

[19] Azimi S.F., Mainella V., Jeffres M.N. (2022). Immediate Hypersensitivity to Fluoroquinolones: A Cohort Assessing Cross-Reactivity. Open Forum Infect. Dis..

[20] Fukushima K., Nakatsubo M., Noda M., Uenami T., Hayama Y., Tsuruta N., Oniki S., Saito Y., Niju T., Ikeda T. (2012). Anaphylaxis Due to Intravenous Levofloxacin with Tolerance to Garenoxacin. Intern. Med..

[21] Shah S., Clarke L.G., Adams K.K. (2023). In-Class Cross-Reactivity among Hospitalized Patients with Hypersensitivity Reactions to Fluoroquinolones. Antimicrob. Agents Chemother..

[22] Dewachtera P., Mouton-Faivreb C. (2013). Anaphylaxis to levofloxacin. Allergol. Immunopathol..

[23] Demir S., Gelincik A., Akdeniz N., Aktas-Cetin E., Olgac M., Unal D., Ertek B., Coskun R., Colakoğlu B., Deniz G. (2017). Usefulness of In Vivo and In Vitro Diagnostic Tests in the Diagnosis of Hypersensitivity Reactions to Quinolones and in the Evaluation of Cross-Reactivity: A Comprehensive Study Including the Latest Quinolone Gemifloxacin. Allergy Asthma Immunol. Res..

